# The Profile of Carotenoids and Other Bioactive Molecules in Various Pumpkin Fruits (*Cucurbita maxima* Duchesne) Cultivars

**DOI:** 10.3390/molecules24183212

**Published:** 2019-09-04

**Authors:** Bartosz Kulczyński, Anna Gramza-Michałowska

**Affiliations:** Department of Gastronomy Sciences and Functional Foods, Faculty of Food Science and Nutrition, Poznań University of Life Sciences, Wojska Polskiego 31, 60–624 Poznań, Poland

**Keywords:** pumpkin fruits, *Cucurbita maxima* Duchesne, bioactive compounds, cluster analysis

## Abstract

Bioactive compounds are significant to human nutrition. They are beneficial to health as they inhibit the development of numerous diseases of affluence. Scientists continuously search for natural sources of these components. At present, the chemical composition of various plants is under investigation. Many researchers are interested in pumpkin (Cucurbita L. spp.). Different organs of this plant (pulp, seeds, flowers, leaves, shoots, roots) are consumed almost all over the world. They contain large amounts of bioactive compounds. Pumpkin pulp is used to prepare various dishes. It is also widely used in the food industry for the production of pastries, baked goods, juices, jams, marinades, and baby food. The content of carotenoids in the pumpkin has been documented in a large number of publications. However, so far there has been no complex analysis of the profile of other bioactive compounds. This article analyses 11 pumpkin cultivars of the *Cucurbita maxima* Duchesne species. It compares the chemical composition of the pulp and analyses the content of bioactive compounds such as carotenoids, polyphenols (flavonols and phenolic acids), tocopherols, minerals (K, Ca, Mg, Na, Fe, Zn, Cu, Mn), vitamins (C, B1, folates). In view of available information, the study provides an innovative approach. The analysis showed high diversity in the concentration of individual components between the cultivars. The research proved that pumpkin pulp was not only a source of carotenoids but also other bioactive compounds.

## 1. Introduction

The diet is unquestionably significant for human health because it inhibits the development of numerous diseases of affluence [1]. Scientists continue searching for appropriate nutritional models with a positive effect on all people’s health. Research on the chemical composition of raw materials and food products consumed in an everyday diet is one of the tools helping to achieve this goal. Identifying the content of individual compounds enables personalization of diets according to consumers’ individual needs. Fruit and vegetables are a very important group of products, which make the base of the food pyramid. They contain a large number of bioactive compounds which are beneficial to health [2]. The definition of bioactive compounds is not fully consistent, but according to many authors, it is a group of physiologically active components of animal and plant origin, which may modify the organism physiology and metabolism when consumed. Simultaneously, they exhibit a wide spectrum of health-promoting effects and they are safe to health [3]. This group mostly includes polyphenols, carotenoids, tocopherols, sterols, stanols, minerals, vitamins, bioactive proteins, peptides, dietary fiber, pre- and probiotics, and fatty acids. These compounds have been proved to have a positive effect on human health.

Many studies have confirmed their inhibition of cardiovascular, gastrointestinal, infectious, and neurodegenerative diseases, as well as their therapeutic effect in carbohydrate metabolism disorders [4,5,6,7]. Pumpkin (*Cucurbita* L.) is a rich source of bioactive compounds. It is an annual plant of the *Cucurbitaceae* family, which includes about 130 genera and more than 800 species [8]. It is believed that the pumpkin is the most diverse vegetable in terms of characteristics such as size, shape, color [9]. Pumpkins blooms from July to September and the seeds ripen from August to October [10]. The origin of the pumpkin has not been clearly identified, but it is supposed to have been first grown in present-day Mexico about 5500 BC. At present pumpkins are common almost all over the world. They can mostly be found in Europe, North and South Americas as well as some regions in Asia (India, China) [11]. In 2013, the global production of pumpkin accounted for 24,679.859 tons. China (7155.250 tons), India (4900.000 tons), and Russia (1128.205 tons) have the largest share in world production. The total area of pumpkin cultivation in India is 1.8 mln hectares, and it is more than three times as large as in the order of China [12]. Nearly all organs of the pumpkin plant (fruits, flowers, leaves, roots, shoots, and seeds) are edible, with some differences between cultivars [13,14]. For example, oils from different pumpkin seeds may have different sensory characteristic (e.g., taste, smell). In addition, it was not clearly stated whether, for example, all decorative pumpkin varieties are edible. However, it is worth to be careful when deciding to consume less known varietes, usually used for decoration. The results of research using squashes show that some varieties may lead to gastrointestinal discomfort (abdominal pain, vomiting, diarrhea). The authors of the experiment recommend not to use all these varieties that have a bitter taste [15]. Before consumption pumpkin is usually boiled, baked, or processed into marinades, juices, jams, etc. It is also used in the food industry as an ingredient of pastries, baked goods, sweets, and baby food [16,17,18].

**Figure 1:**
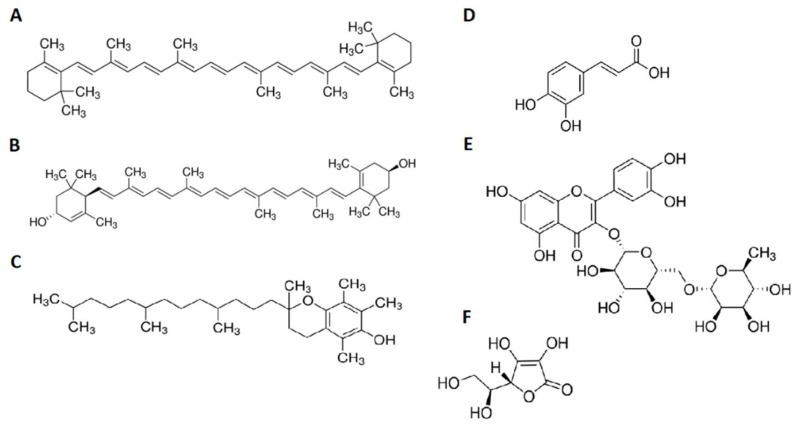
Chemical structure of selected bioactive compounds found in pumpkin flesh (**A**—β-carotene, **B**—lutein, **C**—α-tocopherol, **D**—caffeic acid, **E**—rutin, **F**—ascorbic acid).

Pumpkin pulp is thought to be a source of carotenoids. So far, most studies on the chemical composition of pumpkin pulp have focused on the content of these compounds [19,20,21,22,23,24]. In spite of the considerable interest in the chemical and health-promoting properties of pumpkin, there have been few studies on the profile of other bioactive compounds, which are important for nutrition [25]. Apart from that, as was mentioned in the Introduction, there are a lot of cultivars within one species. Therefore, this study attempts to make a comparative analysis of the content of bioactive compounds (carotenoids, polyphenols, tocopherols, minerals, vitamins) in 11 fruits of pumpkin cultivars (*C. maxima* Duchesne). Chemical structures of some bioactive compounds found in pumpkin fruits are shown in Figure 1.

## 2. Results and Discussion

### 2.1. Bioactive Compounds Content

Carotenoids are an important group of bioactive compounds, which are ascribed a broad spectrum of health-promoting effects. So far research has proved high diversity in carotenoid concentration in different *Cucurbitaceae* species and cultivars. Table 1 shows the content of individual carotenoids in the pumpkin cultivars under study. We chose these carotenoids, which are most often discussed in the literature in terms of the pumpkin chemical composition. The carotenoids under analysis (zeaxanthin, lutein, and β-carotene) were found in all the cultivars. There were statistically significant differences between all the cultivars in the content of zeaxanthin and lutein. The highest content of these carotenoids was found in the ‘Melonowa Żółta’ (zeaxanthin: 192.5 µg/g, lutein: 388.79 µg/g) and ‘Green Hubbard’ cultivars (zeaxanthin: 103.87 µg/g, lutein: 239.38 µg/g). The lowest content of zeaxanthin was found in the ‘Buttercup’ cultivar (19.57 µg/g), whereas the lowest content of lutein was measured in the ‘Porcelain Doll’ cultivar (87.2 µg/g). The highest content of β-carotene was found in the ‘Melonowa Żółta’ (115.29 µg/g) and ‘Jumbo Ping Banana’ cultivars (102.45 µg/g), whereas the lowest content was measured in the ‘Porcelain Doll’ (38.67 µg/g), ‘Hokkaido’ (40.10 µg/g) ‘Marina di Chioggia’ (47.17 µg/g), and ‘Jarrahdale’ cultivars (49.38 µg/g). Lutein was the predominant carotenoid in all the cultivars. The results were used to calculate the retinol equivalent—a measure of the provitamin A content. The highest content of provitamin A was found in the ‘Melonowa Żółta’ (67.66 µg/g), ‘Green Hubbard’ (43.54 µg/g) and ‘Jumbo Pink Banana’ cultivars (41.68 µg/g), whereas the lowest content was measured in the ‘Porcelain Doll’ (15.9 µg/g) and ‘Marina di Chioggia’ cultivars (22.2 µg/g).

Seleim et al. analyzed the content of β-carotene in the ‘Faraskour’, ’El-Zarka’ and ‘Kafr El-Battikh-2’ cultivars of the *C. maxima* Duchesne species and proved significant differences. The researchers noted the highest β-carotene concentration in the ‘Kafr El-Battikh-2’ cultivar (3313.46 µg/100 g). However, it was lower than the concentration measured in the cultivars analyzed in our study. Norshazila et al. [22] studied cultivars of the *Cucurbita moschata* species and observed that β-carotene was the predominant carotenoid. The cultivars also contained small amounts of lutein and α-carotene. We made different observations in our study, where lutein was the most abundant carotenoid. The different proportions of lutein: beta-carotene may be influenced mainly by two factors. The first is the type of variety. For example, in the ‘Buttercup’ variety the ratio of lutein, beta-carotene is close to 1:1. In turn for the ‘Melonowa Żółta’ variety, the ratio is over 3:1. Secondly, the proportion of lutein: beta-carotene can also be affected by the growing conditions, the maturation period, and the storage time of the pumpkin. It seems that during the growth and maturation of the plant, as well as the storage of fruit, the conversion of individual carotenoids may proceed at different rates. As Jaswir et al. showed that during pumpkin fruits storage, the concentration of lutein increases, while beta-carotene decreases. The changes in carotenoids also depend on sun exposure, temperature, water availability, and soil composition. It is believed that the highest concentration of carotenoids occurs in the first stages of fruit formation. After their maturation, the content of these compounds is significantly reduced. However, the directions of these changes are not examined in detail and may constitute an interesting and new purpose of research [26]. Seven cultivars of three species (*C. maxima* Duchesne, *C. moschata*, *C. pepo* L.) were characterized by high diversity in the content of β-carotene. The highest content of carotenoids was found in the ‘Kroshka’ (6.59 mg/100 g, *C. maxima* Duchesne), ‘Zemcuzina’ (5.30 mg/100 g, *C. moschata*) and ‘Bambino’ cultivars (4.50 mg/100 g, *C. maxima* Duchesne), whereas the lowest content was measured in the ‘Zalushka’ (0.30 mg/100 g, *C. pepo* L) and ‘Kustovaja’ cultivars (0.58 mg/100 g, *C. pepo* L). The β-carotene concentration in the ‘Kroshka’ and ‘Zemcuzina’ cultivars was similar to the content measured in cultivars analyzed by other researchers, e.g., ‘Gomez’, ‘Blue Kuri’, ‘Jarrahdale’ [20]. Kulaitienė et al. [21] analyzed the ‘Justynka F1’, ‘Karowita’, ‘Amazonka’ cultivars of the *C. maxima* Duchesne species and found that lutein and zeaxanthin were the most abundant carotenoids (7.96–12.31 mg/100 g).

There were similar results in our study, where lutein and zeaxanthin were the predominant carotenoids [21]. The content of β-carotene ranged from 1.86 to 2.42 mg/100 g and it was lower than the content of this carotenoid measured in the cultivars analyzed in our study. The samples also contained lycopene (0.72–0.81 mg/100 g). Azevedo-Meleiro et al. [19] found different carotenoids, depending on the pumpkin species and cultivar (Azevedo-Meleiro and Rodriguez-Amaya, 2007). The ‘Menina Brasileira’ and ‘Goianinha’ cultivars (*C. moschata*) were characterized by high content of α-carotene (23.8–26.8 µg/g) as well as β-carotene (56.7–66.7 µg/g), like the ‘Jarrahdale’ (49.38 µg/g), ‘Gomez’ (51.28 µg/g) and ‘Blue Kuri’ (56.03 µg/g) cultivars analyzed in our study. There were smaller amounts of lutein and neoxanthin. The ‘Exposicao’ cultivar had the highest content of violaxanthin (20.6 µg/g). The ‘Tetsukabuto’ cultivar (*C. maxima* Duchesne *× C. moschata* hybrid) contained the most lutein (56.6 µg/g), but its content was lower than the content of this carotenoid measured in all the cultivars analyzed in our study (87.2–388 µg/g).

Table 1 shows the content of tocopherols. The pumpkin cultivars analyzed in our study contained two forms: α- and γ-tocopherol, but none of them contained δ- or β-tocopherol. The ‘Melonowa Żółta’ cultivar had the highest content of α-tocopherol (8.89 mg/100 g). The ‘Gomez’ and ‘Marina di Chioggia’ cultivars also contained high amounts of this compound (4.94 mg/100 g and 4.39 mg/100 g, respectively). The lowest concentration of α-tocopherol was measured in the following cultivars: ‘Jumbo Pink Banana’ (1.55 mg/100 g), ‘Hokkaido’ (2.82 mg/100 g) and ‘Jarrahdale’ (2.85 mg/100 g). Similarly, to the content of α-tocopherol, the ‘Melonowa Żółta’ cultivar was characterized by the highest concentration of γ-tocopherol (17.32 mg/100 g). The lowest concentration of γ-tocopherol was measured in the following cultivars: Jarrahdale” (0.97 mg/100 g), ‘Porcelain Doll’ (1.6 mg/100 g) and ‘Bambino’ (2.8 mg/100 g). The contents of individual tocopherols were used to calculate the α-tocopherol equivalent. The highest values of the α-tocopherol equivalent were measured in two cultivars, i.e., ‘Melonowa Żółta’ (10.62 mg/100 g) and ‘Hokkaido’ (5.29 mg/100 g). The lowest values of the α-tocopherol equivalent were measured in the ‘Jumbo Pink Banana’ (2.19 mg/100 g) and ‘Jarrahdale’ cultivars (2.94 mg/100 g).

Like in our study, Seleim et al. [23] observed that the pulp of pumpkins of the *C. maxima* Duchesne species contained α-tocopherol. The authors of the study found that the ‘El Zarka’ cultivar had the highest content of this component (1547.35 µg/100 g). However, it was smaller than the content of α-tocopherol measured in all the cultivars analyzed in our study and it was very similar to the content measured in the ‘Jumbo Pink Banana’ cultivar (1.55 mg/100 g). The lowest content of α-tocopherol was found in the ‘Kafr El-Battikh-2’ cultivar (774.52 µg/100 g). The statistical analysis also confirmed that the content of this component in pumpkin pulp was significantly greater than in pumpkin skin. There are no data on the content of individual tocopherols in pumpkin pulp. Stevenson et al. [27] characterized the profile of these compounds in oil extracted from pumpkin seeds. They observed the highest content of α-tocopherol in the following cultivars: ‘Warren Scarlet’ (75.1 µg/g), ‘Big Max’ (61.3 µg/g) and ‘Zapallo Macre’ (57.6 µg/g). Our study showed that the pulp of the ‘Melonowa Żółta’ cultivar was the most abundant in α-tocopherol (8.89 mg/100 g). The other cultivars contained smaller amounts of this compound (1.55–4.94 mg/100 g) [27]. There was low content of α-tocopherol in pumpkin seed oil extracted from the following cultivars: ‘Delica’ (27.1 µg/g), ‘Yogorou’ (29.6 µg/g) and ‘Cha Cha’ (29.9 µg/g). As far as γ-tocopherol is concerned, the ‘Big Max’ (492.8 µg/g), ‘Rouge Vif d’Etampes’ (285.7 µg/g) and ‘Yogorou’ cultivars (227.0 µg/g) were the most abundant in this compound. There was considerable diversity between the cultivars in their content of δ-tocopherol, which ranged from 35.3 to 1109.7 µg/g. The highest content of this compound was found in the ‘Kurijiman’ cultivar. We did not find δ-tocopherol in the pumpkin pulp in our study. Ryan et al. compared the content of tocopherols in different seeds, cereals, and leguminous plants [28]. They proved that pumpkin seeds contained more α-tocopherol (0.9 mg/100 g) than butter beans (0.7 mg/100 g), spelt (0.6 mg/100 g), buckwheat (0.1 mg/100 g), maize (0.2 mg/100 g), millet (0.2 mg/100 g), linseed (0.1 mg/100 g), mustard (0.6 mg/100 g), and poppy (0.2 mg/100 g). Apart from that, the research proved that pumpkin seeds contained the largest amounts of β + γ-tocopherol (14.8 mg/100 g).

The analysis of the content of phenolic acids confirmed high diversity between the pumpkin cultivars (Table 2). This work focuses on the determination of the selected phenolic compounds content. For the study, phenolic acids and flavonols, which are most often analyzed in plant-derived products and discussed in literature were selected. The ‘Melonowa Żółta’ cultivar had the highest content of gallic acid (18.90 mg/100 g) and protocatechuic acid (47.89 mg/100 g). The highest content of *p*-coumaric acid was found in the ‘Hokkaido’ cultivar (3.47 mg/100 g), and the highest content of ferulic acid was found in the ‘Melonowa Żółta’ cultivar (33.50 mg/100 g). The lowest content of 4-hydroxybenzoic acid (4.95 mg/100 g) and ferulic acid (6.87 mg/100 g) was found in the ‘Green Hubbard’ cultivar. The ‘Bambino’ cultivar was characterized by the lowest concentration of gallic acid (2.58 mg/100 g) and protocatechuic acid (5.09 mg/100 g). Apart from that, it did not contain phenolic acids or *p*-coumaric acid, which was also the case with the ‘Gomez’ cultivar. The ‘Jarrahdale’, ‘Buttercup’, ‘Porcelain Doll’, and ‘Gomez’ cultivars did not contain any sinapic acid. 

The analysis of the samples showed diversified content of flavonols (Table 2). All the cultivars contained rutin. The highest content of this compound was found in the ‘Bambino’ cultivar (51.92 mg/100 g), whereas the lowest content was measured in the ‘Marina di Chioggia’ cultivar (5.09 mg/100 g). The ‘Bambino’ cultivar was also characterized by the highest concentration of astragalin (28.03 mg/100 g) and myricetin (9.04 mg/100 g). Some pumpkin cultivars did not contain kaempferol (‘Bambino’, ‘Porcelain Doll’, ‘Buttercup’, ‘Jarrahdale’), astragalin (‘Hokkaido’, ‘Gomez’, ‘Buttercup’, ‘Jumbo Pink Banana’, ‘Green Hubbard’), myricetin (‘Gomez’, ‘Melonowa Żółta’, ‘Jarrahdale’, ‘Marina di Chioggia’), or isoquercetin (‘Buttercup’, ‘Green Hubbard’). The ‘Jumbo Pink Banana’ cultivar did not contain quercetin. There are few publications on the profile of phenolic compounds in pumpkin fruit.

Dragovic-Uzelac et al. observed chlorogenic acid (15.59–23.05 mg/kg) in all pumpkin cultivars under study [29]. This content was similar to the one measured in the cultivars analyzed in our study: ‘Gomez’ (1.39 mg/100 g), ‘Jarrahdale’ (1.94 mg/100 g) and ‘Marina di Chioggia’ (2.45 mg/100 g). All the cultivars contained syringic acid (19.56–27.13 mg/kg) (Dragovic-Uzelac et al., 2005). Two cultivars, i.e., ‘Turkinja’ (*C. maxima* Duchesne) and ‘Argenta’ (*C. moschata*) contained small amounts of caffeic acid (1.05 and 2.33 mg/kg, respectively), which were smaller than the content of caffeic acid measured in all the cultivars analyzed in our experiment. Only the ‘Argenta’ cultivar contained *p*-coumaric acid (1.27 mg/kg). This acid was identified in nearly all the cultivars in our study, except ‘Bambino’ and ‘Gomez’. Zdunic et al. identified eight polyphenolic compounds in pumpkin fruit (*C. maxima* Duchesne): protocatechuic acid, chlorogenic acid, salicylic acid, p-hydroxybenzoic acid, *p*-coumaric acid, hesperidin, vanillin, and eriodictyol-7-neohesperidoside [30]. The researchers found that the total content of polyphenols amounted to 905.9 µg/g. Biesiada et al. [31] measured the highest content of phenolic compounds in the following pumpkin cultivars: ‘Amazonka’ (*C. maxima* Duchesne, 29.82 mg/100 g), ‘Jet F1’ (*C. pepo* L., 24.90 mg/100 g), and ‘Junona’ (*C. pepo* L, 23.10 mg/100 g). The smallest content of these compounds was identified in the ‘Melonowa Żółta’ (*C. maxima* Duchesne, 14.75 mg/100 g), ‘Ambar’ (*C. maxima* Duchesne, 18.05 mg/100 g) and ‘Pyza’ cultivars (*C. pepo* L, 18.18 mg/100 g) [31]. Telesiński et al. observed that there were statistically significant differences (*p* < 0.05) in the total content of polyphenols and flavonoids between pumpkin cultivars (‘Kurinishiki F1’, ‘Butternut Rugosa’, ‘Muscade de Provence’, ‘Muskatna’) of the *C. moschata* species [32]. The authors proved that the ‘Kurinishiki F1’ cultivar had the highest content of total polyphenols (998.63 mg/kg), whereas the ‘Muscade de Provence’ cultivar contained the smallest amount of these compounds (737.02 mg/kg). The ‘Kurinishiki F1’ cultivar also had the highest content of total flavonoids (725.29 mg/kg). The lowest content of total flavonoids was measured in the ‘Butternut Rugosa’ cultivar (548.36 mg/kg). Altemimi et al. found that pumpkin fruit contained ellagic acid (2.96 µg/g) and myricetin (2.95 µg/g) [33].

The research results show that there was considerable diversity between the cultivars in their content of minerals (Table 3). The highest content of potassium was found in the ‘Bambino’ cultivar (9965.7 mg/100 g). The concentration of this mineral was slightly different in the ‘Jumbo Pink Banana’ (8306.43 mg/100 g) and ‘Marina di Chioggia’ cultivars (7441.2 mg/100 g). The following cultivars were the most abundant in calcium: ‘Melonowa Żółta’ (264.89 mg/100 g), ‘Marina di Chioggia’ (241.91 mg/100 g), ‘Jumbo Pink Banana’ (228.6 mg/100 g) and ‘Porcelain Doll’ (217.01 mg/100 g). The smallest amount of calcium was found in the ‘Hokkaido’ cultivar (92.12 mg/100 g). There was a high content of magnesium measured in the ‘Bambino’ (135.35 mg/100 g) and ‘Marina di Chioggia’ cultivars (127.91 mg/100 g). The largest amounts of copper and manganese were measured in the Bambino’ cultivar (0.59 mg/100 g and 0.86 mg/100 g, respectively). The highest concentrations of iron and zinc were measured in the ‘Melonowa Żółta’ cultivar (2.67 mg/100 g and 1.33 mg/100 g, respectively). The content of sodium in the pumpkin cultivars ranged from 226.16 to 370.40 mg/100 g. The highest content was found in the ‘Porcelain Doll’ cultivar, whereas the lowest content was measured in the ‘Buttercup’ cultivar. The data provided by the United States Department of Agriculture Food Composition Databases (USDA, 2015) indicate that potassium is the most abundant mineral in pumpkin. This statement is in agreement with the results of our study. The content of potassium varied between the cultivars as follows: ‘Butternut’ (352 mg/100 g), ‘Hubbard’ (320 mg/100 g), ‘Spaghetti’ (108 mg/100 g) [34]. Blessing et al. also noted low amounts of iron, calcium, and phosphorus in their study [35]. The contents of these elements ranged from 0.0069 to 0.136 mg/100 g (iron), from 0.461 to 0.610 mg/100 g (calcium), and from 0.145 to 1.093 mg/100 g (phosphorus) [35]. Elinge et al. analyzed the content of minerals in pumpkin seeds and found that like in pumpkin pulp, potassium was the most abundant element (237.24 mg/100 g) [36]. Apart from that, pumpkin seeds contained relatively large amounts of sodium (170.35 mg/100 g), magnesium (67.41 mg/100 g) and zinc (14.14 mg/100 g). They also contained small amounts of iron (3.75 mg/100 g), calcium (9.78 mg/100 g) and manganese (0.06 mg/100 g).

There were statistically significant differences in the content of vitamins between the pumpkin cultivars (Table 3). The highest content of vitamin C was found in the ‘Melonowa Żółta’ cultivar (84.23 mg/100 g). There were slightly smaller amounts of this vitamin in the ‘Jarrahdale’ (80.94 mg/100 g) and ‘Porcelain Doll’ cultivars (71.84 mg/100 g). The lowest concentration of vitamin C was measured in the ‘Hokkaido’ (49.16 mg/100 g) and ‘Buttercup’ cultivars (49.06 mg/100 g). The highest amounts of thiamine were found in the ‘Hokkaido’ (0.6 mg/100 g), ‘Jumbo Pink Banana’ (0.54 mg/100 g) and ‘Jarrahdale’ cultivars (0.52 mg/100 g). The smallest amount of vitamin B1 was measured in the ‘Blue Kuri’ cultivar (0.15 mg/100 g). There was only one form of folates in the samples, i.e., 5-methyltetrahydrofolate (5CH3FH4). There was no tetrahydrofolate (FH4) or 5-formyltetrahydrofolic acid (5-HCO-FH4). The highest concentration of folates was measured in the ‘Marina di Chioggia’ (65.04 ± 0.88 µg/100 g), ‘Bambino’ (52.10 µg/100 g) and ‘Melonowa Żółta’ cultivars (50.99 µg/100 g). The lowest content of these compounds was found in the ‘Jarrahdale’ (20.44 µg/100 g) and ‘Hokkaido’ cultivars (22.46 µg/100 g). The content of vitamin C in different pumpkin cultivars was analyzed in a few studies. Assous et al. [37] found that the pumpkin of the *C. moschata* species contained L-ascorbic acid (33.81 mg/100 g of fresh weight). The content was smaller than in our study (49.16–80.94 mg/100 g of dry mass). Offor et al. noted a low concentration of this compound (4.93 mg/100 g), which was similar to that of cabbage (4.40 mg/100 g) [38]. Nawirska-Olszańska et al. observed that the content of vitamin C in the ‘Karowita’ pumpkin cultivar (*C. maxima* Duchesne) amounted to 14.12 mg/100 g [39]. Biesiada et al. [31] observed considerable diversity between the cultivars in their content of ascorbic acid. The highest concentration of this compound was measured in the following cultivars: ‘Ambar’ (42.48 mg/100 g), ‘Amazonka’ (34.97 mg/100 g), ‘Karowita’ (29.18 mg/100 g), and ‘Bambino’ (26.05 mg/100 g). The ‘Jet F1’, ‘Junona’ and ‘Miranda’ cultivars contained small amounts of ascorbic acid, i.e., 10.80, 9.22, and 5.54 mg/100 g, respectively. The study also proved that the content of this compound decreased as the storage time increased. After 90 days of storage, the concentration of vitamin C dropped by about 40–50%. Sharma and Rao [40] proved that the content of vitamin C differed according to the phase of maturity.

The longer the maturity period was, the more ascorbic acid was found. The content of ascorbic acid in young fruits was 4.30 mg/100 g, whereas fully mature fruits contained 15.00 mg/100 g. The data provided by the USDA [34] also indicated differences between cultivars in the content of vitamin C. The content of vitamin C in the ‘Butternut’ cultivar amounted to 21.0 mg/100 g. The concentration of ascorbic acid in other cultivars was lower, i.e., 11.0 mg/100 g (‘Acorn’, ‘Hubbard’) and 2.1 mg/100 g (‘Spaghetti’). The highest content of thiamine was measured in the ‘Acorn’ (0.140 mg/100 g) and ‘Butternut’ cultivars (0.100 mg/100 g). There were smaller amounts of vitamin B1 in the ‘Hubbard’ (0.070 mg/100 g) and ‘Spaghetti’ cultivars (0.037 mg/100 g). The largest number of folates was found in the ‘Butternut’ cultivar (27 µg/100 g), whereas the smallest amount was noted in the ‘Spaghetti’ cultivar (12 µg/100 g) [34].

### 2.2. Correlation of Bioactive Compounds Content of Different C. maxima Duchesne Varieties

The correlation analysis indicated a wide range of dependencies between the contents of individual compounds exhibiting antioxidative properties (Table 4). The concentrations of phenolic acids and iron exhibited the strongest positive correlation (r = 0.68, *p* < 0.001). The high concentration of iron was also related to the high content of zinc and manganese (r = 0.45, *p* < 0.05). There were also high values of the correlation coefficients between the content of tocopherols and carotenoids (r = 0.50, *p* < 0.01) as well as between the content of tocopherols and flavonols (r = 0.50, *p* < 0.01). There was a strong positive correlation between the concentration of flavonols and the content of copper (r = 0.65, *p* < 0.001). There were negative correlations between the content of phenolic acids and copper (r = −0.42, *p* < 0.05) and between the content of tocopherols and manganese (r = −0.41, *p* < 0.05). There were no statistically significant correlations between the content of vitamin C and the other compounds analyzed in the study. It can be assumed that both positive and negative correlations between the content of the bioactive compound may result from their antioxidative and prooxidative activity. Bioactive compounds have a certain synergistic effect in terms of antioxidant properties and thereby prevent their reduction. However, the high content of antioxidants may also intensify prooxidative modifications leading to the destruction of the bioactive compounds structure.

### 2.3. Cluster Analysis

**Figure 2:**
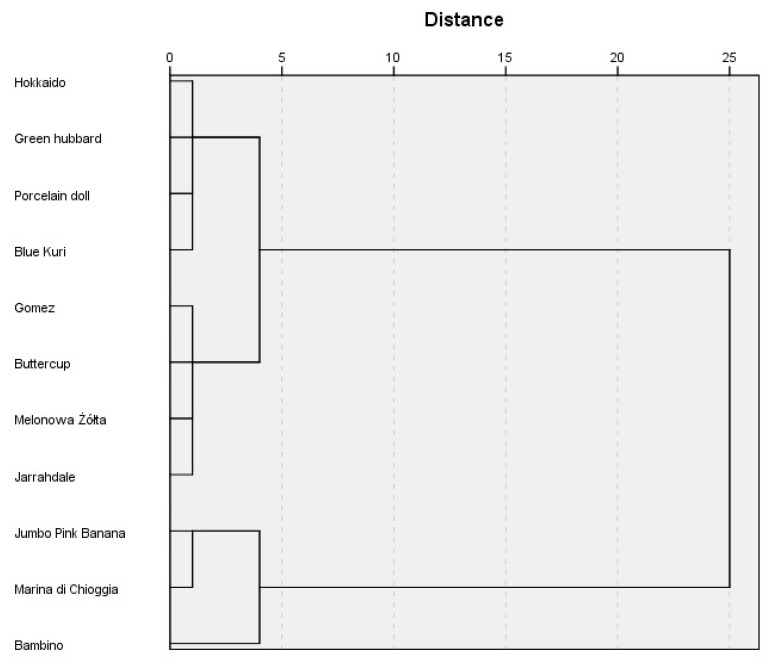
Dendrogram of studied pumpkin varieties division.

We conducted cluster analysis to identify pumpkin cultivars according to the content of bioactive compounds. We assumed that the groups identified in the analysis should differ according to the most determinative variables. We conducted hierarchical cluster analysis using Ward’s method. The number of groups and the cultivars belonging to these groups were identified according to a dendrogram (Figure 2). There were two clearly different groups of cultivars identified according to bioactive compound contents: Cluster 1 (*n* = 3): ‘Bambino’, ‘Jumbo Pink Banana’,‘Marina di Chioggia’, Cluster 2 (*n* = 8): ‘Hokkaido’, ‘Gomez’, ‘Melonowa żółta’, ‘Blue Kuri’, ‘Buttercupi’, ‘Jarrahdale’, ‘Green Hubbard’, ‘Porcelain Doll’. Next, the groups were analyzed with Student’s t-tests for independent samples to identify the variables causing differences between the groups. The analysis revealed (Table 5) that there were statistically significant differences between the groups in the content of sinapic acid, quercetin, potassium, magnesium, iron, vitamin C, and folates. The first group contained statistically larger amounts of sinapic acid (23.41 vs 8.2 mg/100 g), potassium (8571.11 vs 5575 mg/100 g), magnesium (125.42 vs. 94.56 mg/100 g), iron (2.14 vs 1.78 mg/100 g) and folates (53.48 vs 35.63 μg/100 g). The other group contained larger amounts of quercetin (13.53 vs 5.02 mg/100 g) and vitamin C (65.54 vs 58.16 mg/100 g). There were no statistically significant differences between the groups in the other variables.

## 3. Materials and Methods

### 3.1. Reagents

Analytical standards (Zeaxanthin, Lutein, β-carotene, Gallic acid, Protocatechuic acid, 4-Hydroxy-benzoic acid, Vanillic acid, Chlorogenic acid, Caffeic acid, *p*-coumaric acid, Ferulic acid, Sinapic acid, Rutin, Kaempferol, Isoquercetin, Astragalin, Myricetin, Quercetin, α-tocopherol, γ-tocopherol, δ-tocopherol, 5-methyltetrahydrofolate, tetrahydrofolate, 5-formyltetrahydrofolic acid, l-ascorbic acid) were obtained from Sigma Aldrich (Poznan, Poland). 10-formylfolic acid and pteroyltri-γ-L-glutamic acid were obtained from Schirck’s Laboratories (Jona, Switzerland). α-amylase obtained from Sigma Aldrich (Poznan, Poland) and γ-glutamyl hydrolase obtained from rat blood plasma (Europa Bioproducts Ltd., Cambridge, UK). Other reagents (acetonitrile, triethylamine, ethyl acetate, pyrogallol, ethanol, potassium hydroxide, orthophosphoric acid, methanol, nitric acid, lanthanum chloride, potassium dihydrogen phosphate) were purchased from Merck (Warsaw, Poland).

### 3.2. Sample Collection and Preparation

Material for the study were 11 fruit varieties of *C. maxima* Duchesne (‘Bambino’, ‘Hokkaido’, ‘Jumbo Pink Banana’, ‘Gomez’, ‘Marina di Chioggia’, ‘Melonowa żółta’, ‘Blue Kuri’, ‘Buttercupi’, ‘Jarrahdale’, ‘Green Hubbard’, ‘Porcelain Doll’), which were purchased at the Cooperative of organic farming products ‘Dolina Mogilnicy’ (Wolkowo, Poland). Material for the study were 11 fruit varieties of C. *maxima* Duchesne (‘Bambino’, ‘Hokkaido’, ‘Jumbo Pink Banana’, ‘Gomez’, ‘Marina di Chioggia’, ‘Melonowa żółta’, ‘Blue Kuri’, ‘Buttercupi’, ‘Jarrahdale’, ‘Green Hubbard’, ‘Porcelain Doll’), which were purchased at the Cooperative of organic farming products ‘Dolina Mogilnicy’ in Wolkowo, Poland. Geographic coordinates of this location are 52°9′23.27″ N, 16°30′10.69″ E. The meteorological data of this location is temperature: 8.9 °C, wind velocity: 3.0 m/s, relative humidity: 78.0%, total annual rainfall—515 mm. During the cultivation of plants, standard care treatments were performed (irrigation, weeding, soil loosening). Pumpkins were collected in October 2016. After harvesting, the fruit was delivered to the laboratory and processed immediately. After washing and cleaning, edible parts (pulp) of the fruit were cut into small pieces and then were freeze-dried (HETO). The dried samples were stored at room temperature, without air and light, until used for further analysis.

### 3.3. Carotenoid Content Analysis

Carotenoids were determined by high-performance liquid chromatography HPLC (with a PDA 2998 detector (Waters). A column RP-18 ODS2 (250 × 4.6 mm, 5 μm) was used. Elution was performed using solvent A (80% acetonitrile with 0.05% trimethylamine), and B (ethyl acetate). A gradient applied was: 65% A:35% B at 35 min., in the next 25 min. up to 50% A:50% B, and then this ratio was maintained in the isocratic system for another 5 min at a flow of 1.0 mL/min. Recordings were performed at a wavelength λ = 450 nm. The identification of compounds was based on spectra in the range from 200 to 600 nm and retention times compared to standards. The concentrations of carotenoids were calculated from the previously prepared calibration curve. In order to determine carotenoid samples were saponified. A sample was weighed into round bottom flasks, followed by addition of pyrogallol, anhydrous ethanol, and potassium hydroxide. After heating the sample, 1% sodium chloride was added, then the sample was cooled and hexane with ethyl acetate was added. After shaking, saturated sodium chloride solution was added. From the upper layer (unsaponifiable matter) volume of 20 mL was collected in a round bottom flask, and the solvent was removed by rotary vacuum evaporator. Then the sample was quantitatively transferred with 2 mL of ethyl acetate to the chromatography vials [41]. Results were expressed as microgram of each carotenoid per gram of dry mass. The retinol equivalent (RA) was calculated according to the following formula: 1 µg retinol equivalent (RA) = 6 µg beta-carotene + 12 µg other carotenoids [42].

### 3.4. Phenolic Compounds Analysis

Quantitative and qualitative analysis of phenolic acids and flavonols in the pumpkin cultivars tested was made using the HPLC technique (Agilent) with an Infinity Bin Pump DAD 1290 detector. The Zorbax SB C18 (3.9 × 150 mm, 5 µm) column was used to determine the free phenolic acids. A non-linear concentration gradient was used in the system: water acidified with orthophosphoric acid (pH 2.7), acetonitrile with water (1:1 *v*/*v*), using a flow rate of 1.5 mL/min. The gradient program started with 100% acidified water and ended with 50% acetonitrile at the 52nd minute of the separation. 10 μL of the sample was injected prior filtration (0.45 μm pore size filter). The identification of the compounds was based on the analysis of standard phenolic acids dissolved in methanol. Identification of individual phenolic acids based on a comparison of the UV-VIS spectra and the acid retention time in samples with the standards determined at λ = 260 and λ = 310 nm. During the flavonols content determination, a non-linear concentration gradient was applied in the system: water acidified with orthophosphoric acid (pH 2.7), acetonitrile with water (1:1 *v*/*v*), using a flow rate of 1.0 mL/min. The gradient program started in the 1st minute from 95% acidified water and it decreased to 20% at the 30th minute [43]. Identification of compounds was performed by comparing their retention times with retention times of the available standards (astragalin, isoquercetin, kaempferol, quercetin, rutin, myrycetin, chlorogenic acid, ferulic acid, gallic acid, caffeic acid, *p*-hydroxybenzoic acid, *p*-coumaric acid, protocatechic acid, sinapic acid, vanillic acid) obtained from Sigma-Aldrich, Poznań, Poland. The content of phenolics in the samples was determined based on prepared calibration curves. Results were expressed as milligram of each polyphenol compound per 100 g of dry mass.

### 3.5. Tocopherols Content Analysis

The tested samples were subjected to saponification. Next, the sample was injected from the upper layer (unsaponifiable substances) for injection by HPLC. Tocopherols were identified qualitatively and quantitatively by HPLC liquid chromatography (Waters 600 Asc. Milford, MA, USA) in a system consisting of a pump Waters 600, column LiChrosorb Si 60 (200 × 4.6 mm, 5 μm, Merck, Darmstadt, Germany) and fluorimetric detector (Waters 474 Asc. Milford MA, USA). The mobile phase was a mixture of n-hexane with 1,4-dioxane (96:4 *v*/*v*). The flow rate was 1.0 mL/min. Detector was set at excitation λ = 290 nm and emission λ = 330 nm. The concentration of individual tocopherols homologues was calculated on the basis of a previously performed calibration curve [44]. Results were expressed as milligram of each tocopherol per 100 g of dry mass.

### 3.6. Mineral Composition Analysis

Determination of mineral components content (Ca, Mg, Mn, Fe, Zn, Cu) was carried out with the atomic-absorption spectrometry method (F-AAS), for the Na and K determination, the atomic-emission spectrometry method was used (F-AES) [45], using the AAS-3 spectrometer (Carl-Zeiss Jena, Germany). To determine the content of minerals, approx. 1 g of the sample was weighed in triplicate. The samples were subjected to prior wet mineralization using spectrally pure, concentrated 65% nitric acid in a closed system using a microwave oven MARS 5 (CEM, USA). The obtained mineralisates were diluted with 1 mol/dm3 HNO3 or 0.3% lanthanum chloride (for Ca and Mg). In the present study, Soya Bean Flour INCTSBF-4 was used as reference material. Results were expressed as milligram of each mineral compound per 100 g of dry mass.

### 3.7. Vitamin C Content Analysis

Separation and identification of vitamin C in tested samples were performed using HPLC high-performance liquid chromatograph (Agilent, Santa Clara, CA, USA) with a detector Infinity Bin Pump DAD 1290. Luna Phenomenex (4.6 × 250 mm, 5 µm) columns were used to separate tested compound. The mobile phase was a mixture of potassium dihydrogen phosphate (Ph = 5,0) and methanol. The volume of injected sample was 20 μL. A gradient from 95% of the potassium dihydrogen phosphate mixture was used in 1 min. up to 78% in 6 min. Identification of vitamin C was based on a comparison of the UV-VIS spectrum and vitamin C retention time in the UV-VIS spectrum, and retention time of the standard. The content of ascorbic acid was determined at λ = 245 nm on the basis of a previously prepared calibration curve [46]. The vitamin C amount was expressed as ascorbic acid, and results were expressed as milligram of vitamin C per 100 g of dry mass.

### 3.8. Thiamine (Vitamin B1) Content Analysis

The thiamine content was determined by using mimetic enzyme iron (III) tetrasulfonatophthalocyanine (FeTSPc). The method is based on the oxidation of thiamine in alkaline medium to give an intensively fluorescent compound [47]. Determination of thiamine content is based on fluorimetric measurement at excitation wavelength λ = 365 nm and using a secondary filter, with a maximum transmittance at a wavelength λ = 435 nm. Results were expressed as microgram of thiamine per 100 g of dry mass.

### 3.9. Folates Content Analysis

*Preparation of standards and enzymes.* Standards (folic acid, 5-methyltetrahydrofolate, tetrahydrofolate) were prepared according to the method described by Konings [48]. α-amylase (E.C. 3.2.1.1, A-6211) was dissolved in 0.1 M phosphate buffer (pH 7.0), directly before the analysis. γ-glutamyl hydrolase was obtained from rat blood plasma (Europa Bioproducts Ltd., Cambridge). *Extraction.* A sample was weighed into the centrifuge tubes and 0.1 M phosphate buffer (pH 7.00) with 2% (*w*/*v*) ascorbic acid and 0.2% (*v*/*v*) 2-merkaptoethanol was added. The solution was intensively mixed and heated in a water bath at 100 °C for 15 min, then cooled in an ice bath to 20 °C, γ-glutamyl hydrolase and α-amylase were added. Subsequently, the samples were incubated at 37 °C for 4 h, then heated at 100 °C for 5 min, and cooled in an ice bath. Samples were centrifuged at 4 °C for 20 min. (12,000 rpm), and the supernatant was decanted into dark glass measuring flasks. 0.1 M phosphate buffer was added to the residual pellet in the tubes, shaken and centrifuged. The supernatant was decanted, and the volume of the flask was refilled with 0.1 M phosphate buffer and filtered.

*Samples purification.* The samples were purified on Bakerbond spe J. T. columns (Baker 7091-03 (quaternary amine)). The separation of the folates was carried out on a liquid chromatograph (Shimadzu LC-10A) equipped with chromatographic column Phenomenex Synergi 4a Hydro-RP 80A (4 µm, 250 × 4.6 mm) [49]. Identification and calculation of folate content were made on the basis of a standard with known folate content. Results were expressed as microgram of folates per 100 g of dry mass.

### 3.10. Statistical Analysis

The results were statistically analyzed using STATISTICA 13.1 (StatSoft, Inc., Cracov, Poland). In order to determine statistically significant differences between varieties, an analysis of variance was made. Multiple comparisons analyses were performed using post-hoc tests NIR. The probability at *p* = 0.05 was assumed as the level of significance. Pearson’s linear correlation coefficients (*p* = 0.05, *p* = 0.01, *p* = 0.001) were calculated between the contents of each compound. A hierarchical cluster analysis was performed using the Ward method, by means of which the pumpkin varieties were grouped in terms of the content of the analyzed components.

## 4. Conclusions

This study compared the chemical composition of the pulp of different pumpkin cultivars (*C. maxima* Duchesne) growing in Poland. So far there has been no study analyzing most of the pumpkin cultivars characterized in this article. Apart from that, there have been few studies analyzing the content of other bioactive compounds than carotenoids in pumpkin pulp. Our experiment had innovative character as it determined a broad profile of bioactive compounds in numerous pumpkin cultivars. The results extended the current knowledge and showed that the cultivars contained large amounts of individual carotenoids (β-carotene, lutein, and zeaxanthin). The cluster analysis confirmed that within one species of pumpkin (C. *maxima* Duchesne) for different varieties there are many significant differences in the content of the bioactive compound. The most significant differences were found for polyphenolic compounds (sinapic acid, quercetin), minerals (potassium, magnesium, iron) and vitamins (vitamin C, folates). These observations are confirmed by other researches. The main factor that may affect such discrepancies are the environmental conditions, mainly weather conditions, the climate zone, cultivation methods (eg fertilizer use, sowing and harvesting time), storage conditions for harvested pumpkins (temperature, time). The analyzed pumpkins were obtained from the same area, which allowed to limit the impact of various environmental conditions on the content of bioactive compounds. Comparing obtained results, it can be concluded that an important factor affecting the content of bioactive compounds in the pumpkin flesh is its genotype (genetic determinants). Such significant variability in the content of the chemical compound is very characteristic for plant materials. 

Therefore, food composition databases (originating from different countries) are based only on average results from various studies. In addition, our research has shown that pumpkin (known for its carotenoids content) is also the source of many other bioactive compounds that have been proven for a health-promoting property. The food market is increasingly offering pumpkin in dried (powdered) form. The results of this study, calculated on a dry weight basis, show that the powdered form of pumpkin also contains many valuable ingredients for our health. Apart from that, the analysis proved considerable diversity between the cultivars in their content of flavonols, phenolic acids, tocopherols, minerals, and vitamins. Diverse bioactive compounds content presented in the tested pumpkin varieties indicates that choosing the right pumpkin variety for consumption significantly affects the number of nutrients supplied. The presence of polyphenols and carotenoids (mainly β-carotene) may probably enhance the antioxidant defense mechanism, preventing the harmful effects of free radicals, which cause many diseases, such as hypertension, atherosclerosis, type 2 diabetes, and cancer. Vitamin C contained in the pumpkin flesh can positively affect immunity and contribute to the increased absorption of iron from the diet. Pumpkin flesh contains high amounts of potassium, which is necessary for the proper functioning of the nervous system and muscles, and also helps to maintain normal blood pressure. The presence of vitamin B1 (thiamine) and B9 (folates) has also been found in pumpkin pulp, but they are present in small amounts, hence they should not be discussed in terms of health-promoting properties. The research results confirmed the high nutritional value of pumpkin and its usefulness for the production of functional food to enhance man health.

## Figures and Tables

**Table 1:** The content of carotenoids and tocopherols in pumpkin varieties.

Cultivars		Bambino	Hokkaido	Gomez	Melonowa Żółta	Porcelain Doll	Blue Kuri	Buttercup	Jumbo Pink Banana	Jarrahdale	Marina di Chioggia	Green Hubbard
Carotenoids (µg/g dm)	Zeaxanthin	53.52 ± 0.44	91.06 ± 1.41	75.44 ± 0.69	192.53 ± 3.30	26.32 ± 0.49	44.17 ± 0.20	19.57 ± 0.44	82.75 ± 1.06	63.72 ± 1.42	48.01 ± 0.82	103.87 ± 4.60
	Lutein	143.02 ± 1.58	130.23 ± 1.30	118.60 ± 0.79	388.79 ± 1.71	87.20 ± 0.73	149.42 ± 2.77	99.05 ± 2.10	212.52 ± 1.60	111.93 ± 2.25	124.08 ± 2.07	239.38 ± 8.48
	β-carotene	86.41 ± 1.35	40.10 ± 0.15 ^a^	51.28 ± 1.15	115.29 ± 0.95	38.67 ± 1.71 ^a^	56.03 ± 0.56	79.61 ± 0.34	102.45 ± 1.43	49.38 ± 1.21	47.17 ± 1.00	89.59 ± 2.60
	Retinol equivalent	30.78 ± 0.17	25.12 ± 0.24 ^a^	24.72 ± 0.29 ^a^	67.66 ± 0.28	15.90 ± 0.36	25.47 ± 0.16 ^a^	23.15 ± 0.27 ^b^	41.68 ± 0.45	22.87 ± 0.49 ^b^	22.20 ± 0.36	43.54 ± 1.42
Tocopherols (mg/100 g dm)	α-tocopherol	4.50 ± 0.05	2.82 ± 0.03 ^a^	4.94 ± 0.03	8.89 ± 0.02	3.70 ± 0.02	3.47 ± 0.04	3.85 ± 0.02	1.55 ± 0.01	2.85 ± 0.01 ^a^	4.39 ± 0.02	4.07 ± 0.04
	γ-tocopherol	2.80 ± 0.03	5.45 ± 0.03 ^a^	3.57 ± 0.11 ^b^	17.32 ± 0.32	1.60 ± 0.02	5.27 ± 0.03 ^a^	3.09 ± 0.01	6.33 ± 0.06	0.97 ± 0.02	3.63 ± 0.09 ^b^	3.36 ± 0.16
	α-tocopherol equivalent	4.78 ± 0.01 ^a^	3.36 ± 0.02	5.29 ± 0.02	10.62 ± 0.03	3.86 ± 0.02	4.00 ± 0.01	4.16 ± 0.02	2.19 ± 0.01	2.94 ± 0.01	4.76 ± 0.02 ^a^	4.41 ± 0.06

^a, b^—means in the same verse followed by the same letters do not significantly differ (*p* < 0.05) in terms of analyzed variables, dm—dry mass, the results are expressed as the mean values ± standard deviation of the triplicate samples, δ-tocopherol and β-tocopherol were not detected in the evaluated samples.

**Table 2:** The content of phenolic acids and flavonols in pumpkin varieties.

	Cultivars	Bambino	Hokkaido	Gomez	Melonowa Żółta	Porcelain Doll	Blue Kuri	Buttercup	Jumbo Pink Banana	Jarrahdale	Marina di Chioggia	Green Hubbard
Phenolic acids (mg/100g dm)	Gallic acid	2.58 ± 0.01	8.05 ± 0.03	6.18 ± 0.09	18.90 ± 0.18	11.44 ± 0.17	8.88 ± 0.08	14.22 ± 0.26	5.93 ± 0.10	12.01 ± 0.18	15.30 ± 0.08	6.90 ± 0.05
	Protocatechuic acid	5.09 ± 0.02	17.38 ± 0.03	8.88 ± 0.04	47.89 ± 0.16	42.13 ± 0.12	18.31 ± 0.06	20.02 ± 0.16	36.81 ± 0.11	6.99 ± 0.05	22.41 ± 0.06	29.82 ± 0.32
	4-Hydroxy-benzoic acid	10.04 ± 0.02	18.01 ± 0.23	7.73 ± 0.03 ^a^	13.2 ± 0.10	33.12 ± 0.18	6.05 ± 0.02	15.36 ± 0.10	7.72 ± 0.03 ^a^	19.13 ± 0.14	22.95 ± 0.09	4.95 ± 0.03
	Vanillic acid	8.81 ± 0.03	6.88 ± 0.04	9.11 ± 0.02	2.03 ± 0.02	6.54 ± 0.03	7.04 ± 0.13	4.20 ± 0.06	3.08 ± 0.07	0.95 ± 0.01	6.34 ± 0.02 ^a^	6.41 ± 0.01 ^a^
	Chlorogenic acid	6.85 ± 0.06	5.12 ± 0.03	1.39 ± 0.21	6.69 ± 0.01	3.06 ± 0.02 ^a^	5.33 ± 0.01	4.03 ± 0.06	1.23 ± 0.01	1.94 ± 0.02	2.45 ± 0.01	3.11 ± 0.01 ^a^
	Caffeic acid	17.75 ± 0.04	48.92 ± 0.02	62.85 ± 0.03 ^a^	126.78 ± 0.05	133.42 ± 0.01	42.38 ± 0.01	36.39 ± 0.01	115.19 ± 0.21	62.70 ± 0.58 ^a^	51.43 ± 0.05	18.97 ± 0.08
	*p*-coumaric acid	n.d	3.47 ± 0.01	n.d	0.79 ± 0.01	1.12 ± 0.01	0.81 ± 0.04	0.90 ± 0.01	2.50 ± 0.01	0.20 ± 0.01	0.54 ± 0.01	0.40 ± 0.02
	Ferulic acid	13.60 ± 0.03	28.90 ± 0.02	9.92 ± 0.03	33.50 ± 0.02	18.07 ± 0.06	18.96 ± 0.1	9.70 ± 0.01	16.00 ± 0.07	24.49 ± 0.03	19.96 ± 0.08	6.87 ± 0.02
	Sinapic acid	32.00 ± 0.08	19.08 ± 0.02	n.d	14.91 ± 0.01	n.d	10.01 ± 0.02	n.d	22.94 ± 0.02	n.d	15.29 ± 0.08	21.58 ± 0.02
Flavonols (mg/100 g dm)	Rutin	51.92 ± 0.03	8.07 ± 0.01	19.90 ± 0.02	30.28 ± 0.06	15.92 ± 0.01	12.10 ± 0.03 ^a^	21.59 ± 0.02	14.05 ± 0.56	6.30 ± 0.02	5.09 ± 0.01	12.10 ± 0.02 ^a^
	Kaempferol	n.d	28.94 ± 0.07	13.10 ± 0.02	20.79 ± 0.01	n.d	15.09 ± 0.01	n.d	7.11 ± 0.02	n.d	13.02 ± 0.05	6.20 ± 0.02
	Isoquercetin	5.54 ± 0.02	5.69 ± 0.01	1.63 ± 0.01	4.11 ± 0.01	9.01 ± 0.09	6.04 ± 0.03	n.d	3.86 ± 0.01	5.33 ± 0.01	4.68 ± 0.17	n.d
	Astragalin	28.03 ± 0.11	n.d	n.d	26.82 ± 0.02	11.39 ± 0.03	15.35 ± 0.03	n.d	n.d	5.40 ± 0.01	12.07 ± 0.03	n.d
	Myricetin	9.04 ± 0.01	1.99 ± 0.02	n.d	n.d	1.74 ± 0.01	6.23 ± 0.01	4.00 ± 0.02	3.83 ± 0.03	n.d	n.d	5.04 ± 0.04
	Quercetin	6.97 ± 0.07	2.55 ± 0.02	26.54 ± 0.02	15.69 ± 0.07	11.03 ± 0.05	18.81 ± 0.07	5.09 ± 0.01	n.d	13.31 ± 0.02	8.10 ± 0.01	15.21 ± 0.02

^a^ means in the same verse followed by the same letter do not significantly differ (*p* *<* 0.05) in terms of analysed variables, dm—dry mass, n.d—not detected, the results are expressed as the mean values ± standard deviation of the triplicate samples.

**Table 3:** The content of mineral compounds and vitamins in pumpkin varieties.

	Cultivars	Bambino	Hokkaido	Gomez	Melonowa Żółta	Porcelain Doll	Blue Kuri	Buttercup	Jumbo Pink Banana	Jarrahdale	Marina di Chioggia	Green Hubbard
Mineral compounds (mg/100g dm)	K	9965.70 ± 0.78	6009.50 ± 1.16	4692.70 ± 1.55	5035.02 ± 0.82	6405.02 ± 0.26	6228.04 ± 1.57	4959.03 ± 0.72	8306.43 ± 1.77	5284.03 ± 1.97	7441.20 ± 2.63	5986.73 ± 3.58
	Ca	106.92 ± 0.10	92.12 ± 0.19	119.08 ± 0.17	264.89 ± 0.59	217.01 ± 0.97	193.74 ± 0.48	169.61 ± 2.05	228.60 ± 0.74	190.11 ± 1.53	241.91 ± 1.74	128.70 ± 1.28
	Mg	135.35 ± 0.08	80.03 ± 0.03 ^a^	111.11 ± 0.19	105.64 ± 0.81	96.25 ± 0.42 ^b^	79.97 ± 2.18 ^a^	96.70 ± 1.21 ^b^	113.00 ± 1.19	101.16 ± 0.21	127.91 ± 1.39	85.63 ± 0.71
	Na	299.33 ± 0.20	292.41 ± 0.42	243.07 ± 1.35	229.94 ± 1.51	370.40 ± 1.88	317.24 ± 1.77 ^a^	226.16 ± 2.12	259.01 ± 0.58	240.04 ± 1.85	317.84 ± 0.43 ^a^	329.76 ± 2.59
	Fe	1.82 ± 0.01	1.55 ± 0.01	1.06 ± 0.01	2.67 ± 0.03	2.25 ± 0.02 ^a^	1.13 ± 0.01	2.22 ± 0.01 ^a^	2.40 ± 0.02	2.10 ± 0.02	2.21 ± 0 ^a^	1.30 ± 0.01
	Zn	1.04 ± 0.01	1.05 ± 0.01	0.84 ± 0.02	1.33 ± 0.01	0.89 ± 0.01	0.87 ± 0.01 ^a^	0.95 ± 0.01	0.87 ± 0.02 ^a^	1.13 ± 0.01	1.18 ± 0.01	0.79 ± 0.01
	Cu	0.59 ± 0.01	0.25 ± 0.01 ^a^	0.52 ± 0.02	0.39 ± 0.01 ^b^	0.28 ± 0.01 ^ac^	0.44 ± 0.01	0.32 ± 0.01 ^cd^	0.29 ± 0.01 ^acd^	0.27 ± 0.01 ^ac^	0.40 ± 0.06 ^b^	0.32 ± 0.01 ^cd^
	Mn	0.86 ± 0.02	0.34 ± 0.01	0.47 ± 0.02 ^a^	0.65 ± 0.02	0.90 ± 0.01	0.48 ± 0.01 ^ab^	0.52 ± 0.01	0.55 ± 0.01	0.49 ± 0.01 ^b^	0.74 ± 0.01	0.81 ± 0.01
Vitamin C (mg/100 g dm)		62.18 ± 0.88 ^a^	49.16 ± 0.77 ^b^	65.17 ± 1.83 ^c^	84.23 ± 1.78	71.84 ± 1.56	63.67 ± 1.77 ^ac^	49.06 ± 0.35 ^b^	60.02 ± 1.72 ^a^	80.94 ± 1.44	52.28 ± 1.22	60.23 ± 2.51 ^a^
Vitamin B1 (mg/100 g dm)		0.29 ± 0.02	0.60 ± 0.01	0.42 ± 0.02 ^a^	0.27 ± 0.01	0.25 ± 0.01	0.15 ± 0.01	0.23 ± 0.01	0.54 ± 0.02	0.52 ± 0.02	0.34 ± 0.01	0.42 ± 0.02 ^a^
Folates (µg/100 g dm)		52.10 ± 1.84 ^a^	22.46 ± 0.49	38.82 ± 1.30	50.99 ± 1.45 ^a^	48.26 ± 0.87	42.04 ± 0.75 ^b^	28.28 ± 0.77	43.31 ± 0.68 ^b^	20.44 ± 0.59	65.04 ± 0.88	33.74 ± 1.81

^a, b, c^—means in the same verse followed by the same letters do not significantly differ (*p<* 0.05) in terms of analyzed variables, K—potassium, Ca—calcium, Mg—magnesium, Na—sodium, Fe—iron, Zn—zinc, Cu—copper, Mn—manganese, dm—dry mass, the results are expressed as the mean values ± standard deviation of the triplicate samples.

**Table 4:** Results of the correlation analysis between the contents of selected compounds with antioxidant properties.

Compound	Sum of Carotenoids	Sum of Phenolic Acids	Sum of Tocopherols	Sum of Flavonols	Vitamin C	Fe	Zn	Cu	Mn
Sum of carotenoids	-	-0.03	0.50 **	0.21	0.12	0.00	−0.08	0.17	0.01
Sum of phenolic acids	−0.03	-	0.23	−0.07	0.33	0.68 ***	0.43 *	−0.42 *	0.06
Sum of tocopherols	0.50 **	0.23	-	0.50 **	0.04	−0.18	0.05	0.43*	−0.41 *
Sum of flavonols	0.21	−0.07	0.50 **	-	0.34	−0.22	0.16	0.65 ***	0.13
Vitamin C	0.12	0.33	0.04	0.34	-	0.14	0.13	0.13	0.17
Fe	0.00	0.68 ***	−0.18	−0.22	0.14	-	0.50 **	−0.31	0.45 *
Zn	−0.08	0.43 *	0.05	0.16	0.13	0.50 **	-	−0.14	0.03
Cu	0.17	−0.42 *	0.43 *	0.65 ***	0.13	−0.31	−0.14	-	0.15
Mn	0.01	0.06	−0.41 *	0.13	0.17	0.45 *	0.03	0.15	-

*p* < 0.05 *, *p* < 0.01 **, *p* < 0.001 ***—determination of statistically significant correlations between tested variables.

**Table 5:** Variable levels divided into groups of pumpkin varieties.

	Compound	Cluster 1	Cluster 2
Carotenoids	Zeaxanthin	61.42 ± 16.19	77.09 ± 52.84
	Lutein	159.87 ± 40.36	165.58 ± 97.21
	β-carotene	78.68 ± 24.66	64.99 ± 25.99
	Retinol equivalent	31.55 ± 8.46	31.05 ± 16.00
Phenolic acids	Gallic acid	7.94 ± 5.71	10.82 ± 4.07
	Protocatechuic acid	21.44 ± 13.76	23.93 ± 14.19
	4-Hydroxy-benzoic acid	13.57 ± 7.11	14.69 ± 8.78
	Vanillic acid	6.08 ± 2.49	5.40 ± 2.64
	Chlorogenic acid	3.51 ± 2.56	3.83 ± 1.72
	Caffeic acid	61.46 ± 42.86	66.55 ± 39.88
	*p*-coumaric acid	1.01 ± 1.14	0.96 ± 1.03
	Ferulic acid	16.52 ± 2.78	18.80 ± 9.24
	Sinapic acid	23.41 ± 7.24 *	8.20 ± 8.95 *
Tocopherols	α-tocopherol	3.48 ± 1.45	4.32 ± 1.88
	γ-tocopherol	4.25 ± 1.60	5.08 ± 4.96
	α-tocopherol equivalent	3.91 ± 1.29	4.83 ± 2.33
Flavonols	Rutin	23.69 ± 21.53	15.78 ± 7.55
	Kaempferol	6.71 ± 5.65	10.52 ± 10.36
	Isoquercetin	4.70 ± 0.73	3.97 ± 3.05
	Astragalin	13.37 ± 12.18	7.37 ± 9.41
	Myricetin	4.29 ± 3.93	2.37 ± 2.34
	Quercetin	5.02 ± 3.80 *	13.53 ± 7.25 *
Mineral compounds	Potassium (K)	8571.11 ± 1111.02 *	5575.00 ± 626.36 *
	Calcium (Ca)	192.48 ± 64.43	171.91 ± 54.20
	Magnesium (Mg)	125.42 ± 9.90 *	94.56 ± 11.17 *
	Sodium (Na)	292.06 ± 26.06	281.13 ± 51.79
	Iron (Fe)	2.14 ± 0.26 *	1.78 ± 0.58 *
	Zinc (Zn)	1.03 ± 0.14	0.98 ± 0.17
	Copper (Cu)	0.43 ± 0.14	0.35 ± 0.09
	Manganese (Mn)	0.72 ± 0.14	0.58 ± 0.18
Vitamins	Vitamin C	58.16 ± 4.65 *	65.54 ± 12.57 *
	Vitamin B1	0.39 ± 0.11	0.36 ± 0.15
	Folates	53.48 ±9.53 *	35.63 ±10.92 *

*p* < 0.05 *—the presence of significant differences between the groups of varieties, the results are expressed as the mean values ± standard deviation of the triplicate samples, the results are expressed in the following units: carotenoids (µg/g dm), phenolic acids, tocopherols, flavonols, mineral compounds (mg/100 g dm), vitamins: C and B1 (mg/100 g dm), folates (µg/100 g dm).
